# Interoceptive profiles of eating and weight disorders

**DOI:** 10.1038/s41598-025-30683-1

**Published:** 2025-12-21

**Authors:** Annika P. C. Lutz, Ann-Kathrin Arend, Ulrich Voderholzer, Claus Vögele, Jens Blechert

**Affiliations:** 1 Department of Behavioural and Cognitive Sciences, Institute for Health and Behaviour, University of Luxembourg, 11 porte des Sciences, 4366 Esch-sur-Alzette, Luxembourg; 2 Department of Psychology, Division of Clinical Psychology and Health Psychology, Paris-Lodron University Salzburg, Salzburg, Austria; 3 Centre for Cognitive Neuroscience, Department of Psychology, Paris-Lodron University Salzburg, Salzburg, Austria; 4 Department of Psychiatry and Psychotherapy, University Hospital of the LMU Munich, Munich, Germany; 5 Department of Psychiatry and Psychotherapy, University Hospital Freiburg, Freiburg, Germany; 6 Schön Klinik Roseneck, Prien am Chiemsee, Germany

**Keywords:** Eating disorders, Obesity, Interoception, Heartbeat-evoked potentials, Interoceptive accuracy, Heart-rate variability, Human behaviour, Obesity, Sensory processing, Obesity, Psychiatric disorders

## Abstract

Signals from the body’s interior undergo several interoceptive processing steps, from the periphery to the central nervous system, to conscious recognition and evaluation. Alterations in some of these steps have been reported for eating and weight disorders, but systematic comparisons between steps and disorders are necessary to develop targeted interventions. We recorded electrocardiogram and electroencephalogram from adult women with anorexia nervosa (AN; *n* = 38), bulimia nervosa (BN; *n* = 35), binge eating disorder (*n* = 22), obesity (OB; *n* = 20), and healthy control individuals (*n* = 46) during resting state and a heartbeat counting task. On the peripheral level, patients with AN, BN, and OB had reduced vagally-mediated heart-rate variability. On the central level, only patients with AN showed increased processing of heartbeats (heartbeat-evoked potentials). On the conscious level, there were no significant group differences in heartbeat-counting performance, beliefs, or insight, but a marked negative evaluation reported by patients with AN and BN. We conclude that fasting could serve to reduce negatively perceived (AN, BN) and increased (AN) bodily sensations. Overeating is not associated with marked alterations in cardiac interoception. The results do not support the notion of general interoceptive deficits, but rather disorder-specific interoceptive profiles.

## Introduction

As early as the 1960’s, it was proposed that alterations in the perception of signals from the body’s interior might be a common factor underlying eating and weight disorders (EWDs)^1^. These range from strict fasting in restrictive anorexia nervosa (AN), to alternating bingeing and purging in bulimia nervosa (BN), to recurrent binge eating in binge eating disorder (BED)^2^, as well as a body mass index (BMI) of 30 or above in obesity (OB)^3^. Recent research was able to identify a range of interoceptive alterations (see e.g.^4^ for a review), suggesting that each EWD might be associated with a unique interoceptive profile. To establish these profiles, it is necessary to investigate a range of interoceptive facets across the spectrum of EWDs.

### Interoception as a Multi-Faceted construct

Interoception is a multi-faceted construct, which refers to the processing of information arising from within the body^5^. Different bodily systems can be distinguished, e.g., the cardio-vascular, gastro-intestinal, or respiratory systems. Altered processing of gastrointestinal information may directly contribute to disordered eating behaviours. Poor perception of gastric fulness has been linked to binge eating, for example^6^. Patients with BN and BED indicate more difficulties perceiving satiety, while patients with AN report more difficulties perceiving hunger^7^. Despite these differences in gastric interoception, all three groups indicated equal difficulties in the perception of emotions^7^. Emotional dysregulation is a common characteristic of EWDs^8^ and may be related to altered processing of cardiovascular information^9^. Both overeating in BN and restrictive eating in AN have been linked to negative emotions and dysfunctional emotion regulation^10^. Fasting in AN may serve to suppress negative emotional states and body sensations^11^. Because of this aetiological relevance and the wide range of available measures, cardiac interoception may be an ideal starting point for the establishment of interoceptive profiles in EWDs. In addition, cardiac interoception could serve as an indicator for common underlying interoceptive factors across bodily systems, especially across the cardiac and gastric modalities^12^. There are several interventions targeting cardioception, including interoceptive exposure^13^, heartbeat perception training^14^, and mindfulness exercises^15^. A better understanding of which aspects of cardiac interoception are altered in which EWDs could guide the selection of targeted interventions and likely improve treatment outcomes.

Several interoceptive facets have been proposed along the processing stream. The current selection of specific facets for the cardiovascular bodily system is based on the recent model proposed by Suksasilp and Garfinkel^16^, spanning physiological, behavioural, and self-report measures.

## Physiological indicators of interoception

### Peripheral organ activity

The activity of the peripheral organ is not considered interoception per se, since the interoceptive process begins with the stimulation of sensory transducers, e.g., mechanoreceptors, but peripheral signal strength may impact higher order interoceptive processing^16^. Regarding the cardiovascular system, the activity of the peripheral organ can be indexed by measures such as heart rate (HR) and parasympathetically mediated high-frequency heartrate variability (HRV)^17^. For example, HR has been reported to correlate positively with heartbeat perception^18^. After short-term fasting, stronger increases in vagally-mediated HRV were related to larger improvements in heartbeat perception^19^. Assessing cardiac activity is especially important in EWDs, since these are commonly associated with cardiovascular alterations, as confirmed by several recent reviews and meta-analyses. Whereas OB is associated with lower HRV^20^, patients with AN and BN tend to show elevated HRV, possibly due to fasting-induced physiological changes^21–23^. There are no marked HRV alterations reported for BED^24^. The relationship between cardiovascular changes in individuals with EWDs and cardiac interoception is not yet well understood.

### Central-Nervous system processing

Heartbeat evoked brain potentials (HEPs) are an electrophysiological indicator of the central-nervous system (CNS) representation of signals from the ﻿heart^25^. They are computed as cortical activity time-locked to the R peak of the electrocardiogram (ECG). Potential sources of the HEP include the insular, cingulate, and somatosensory cortices^26^. They are related both to cardiodynamics and psychological factors such as attention^27^. Stimulation of the vagus nerve^28^ and short-term fasting^29^ have been shown to increase HEPs, yet the potential impact of cardiovascular alterations in EWDs remains unexplored. Additionally, focusing on one’s heartbeat leads to increases in HEP positivity^30^. Investigations of HEPs in EWDs remain scarce and indicate increased processing in AN^31^ and no significant difference in BN^32^, although a recent report suggests findings may depend on analysis choices^33^.

### Self-Report and behavioural indicators of interoception

#### Interoceptive accuracy Performance, Beliefs, and insight

At the cognitive and behavioural levels, heartbeat perception tasks allow for the assessment of several facets of interoceptive accuracy, that is, how accurately participants perceive their own heartbeat^16^. This includes individuals’ *performance* in the task (e.g., correspondence between actually measured and silently counted heartbeats), but also their *beliefs* regarding their own performance^34^. If participants perceive their heartbeat accurately and believe in having performed accurately, they are said to have good interoceptive *insight*, i.e., the correspondence between performance and beliefs^5^.

Heartbeat perception *performance* seems to be reduced in OB^35^. Findings for AN are mixed, with some studies reporting poorer performance in AN than HC (e.g.^36^), and others reporting no significant difference (e.g.^31^). For BN, one study reported reduced heartbeat perception performance in recovered BN^37^, whereas other studies found no significant difference between BN and HC (e.g.^32^). Heartbeat perception *beliefs* have rarely been investigated, with the few extant studies showing reduced or unaltered confidence in their heartbeat perception performance in AN^31, 38^ and no significant effects in BN^32^. We are unaware of any studies addressing heartbeat perception in BED.

#### Emotional evaluation

In addition, negative affect when focusing on one’s heartbeat, that is, the *emotional evaluation*, may play a role in EWDs^39^, considering previous reports of negative affect in response to gastric sensations during a water load test in patients with BN and BED^6^.

Some interoceptive facets have previously been investigated in some EWDs, but there are, to date, no systematic comparisons across facets and EWD diagnoses.

**Table 1:** Evidence gap map for laboratory-based assessment of cardiac interoception in eating and weight disorders.

Interoceptive facet	Measure used	Previous findings			
		AN	BN	BED	OB
Peripheral organ	HF HRV	>	>	=	<
CNS processing	HEPs	>	=	?	?
Accuracy - performance	HCT score	</=	</=	?	<
Accuracy - beliefs	HCT confidence rating	</=	=	?	?
Accuracy - insight	Correspondence HCT score - confidence	?	?	?	?
Emotional evaluation	HCT questionnaire	?	?	?	?

Note. AN = anorexia nervosa; BN = bulimia nervosa; BED = binge eating disorder; OB = obesity; HF HRV = high frequency heartrate variability; CNS = central nervous system; HEPs = heartbeat evoked potentials; HCT = heartbeat counting task.

> higher values in clinical group than control group; < lower values in clinical group than control group; = no significant group difference; ? missing evidence.

#### Hypotheses

In summary, of the extant laboratory-based investigations of interoception in EWDs, the majority examined HRV or heartbeat perception performance, with major gaps in evidence regarding heartbeat perception beliefs, insight, and emotional evaluation, as well as CNS processing of cardioafferent signals (see Table 1 for an evidence gap map). Our hypotheses include, regarding peripheral organ system activity, (1) increased HRV in AN and BN, (2) decreased HRV in OB, and (3) unaltered HRV in BED. On the level of CNS processing, we expected (4) increased HEPs in AN and (5) unaltered HEPs in BN. For heartbeat perception, we expected (6) reduced performance in OB and (7) reduced or unaltered performance in AN and BN, as well as (8) reduced or unaltered beliefs in AN and (9) unaltered beliefs in BN.

## Methods

### Participants

Female participants were recruited at Schön Klinik Roseneck (Prien, Germany) and the University of Salzburg (Austria) in the context of a larger project (see Supplementary Table S2 for a full list of references). For the current manuscript, a subset of *N* = 161 participants was analysed, who had completed one or more measures of interoception and did not meet any of the following criteria: neurological disorders, cardiovascular disease, pregnancy, skin or food allergies, current substance abuse or dependence, age < 18 years. Five groups were investigated: normal-weight healthy control participants (HC; BMI 18.5–20.49) without current mental disorder or lifetime ED, diabetes, or vegetarian/vegan diet; participants currently meeting AN, BN, or BED DSM-5 diagnostic criteria^2^; obese participants (OB; BMI ≥ 30) without current or lifetime ED. The AN and HC samples partially overlap with the samples of Richard et al.^40^, who also reported interoceptive accuracy performance; this is the only overlapping dependent variable, however. Mental disorders were diagnosed with the German Structured Clinical Interview for DSM-IV (SCID-I)^41^, and a shortened German version of the Eating Disorder Examination (EDE)^42^, both adapted to DSM-5 criteria for EDs^2^. The diagnostic interviews were carried out in a separate session before participation in the laboratory session. Trait anxiety was assessed with the German version of the State-Trait Anxiety Inventory, trait version (STAI-T)^43^, a 20-item questionnaire with a scale of 1 *almost never* to 4 *almost always*. The Dutch Eating Behavior Questionnaire (DEBQ)^44^ was used to assess restrained, emotional, and external eating behaviour with 33 items scored 1 *never* to 5 *very often*.

### Procedure

Laboratory sessions took place at 3 pm. Participants tested on campus (HC, OB, BED) were instructed to consume one of several standardised lunch options (~ 550 kcal) at noon. Hospitalized patients (AN, BN) had their regular lunch around noon (~ 700 kcal for underweight patients). The session began with questionnaires and preparation of the sensors, followed by a 4-minute resting-state measurement (one minute each eyes open – closed – open – closed; order counterbalanced across participants), the heartbeat counting task (HCT), and food-picture viewing and decision-making tasks reported elsewhere (see Supplementary Table S2 for a full list of references). The study was approved by the ethics committees of the Universities of Salzburg, Austria, and Munich, Germany. All procedures were in accordance with the Declaration of Helsinki and participants provided written informed consent.

### Heartbeat counting task

Participants were instructed to silently count their heartbeats without taking their pulse, and to only count heartbeats that they actually felt, without trying to guess^45^. After a practice interval of 25 s, there were six randomised counting intervals of 25, 35, 45, 55, 65, and 75 s, followed by prompts to enter the number of counted heartbeats and to rate the confidence in one’s own performance from 1 *not at all sure* to 9 *very sure*. Scores on the HCT (interoceptive accuracy performance) were calculated as 1/6 Σ [1 – (|recorded heartbeats – counted heartbeats|/recorded heartbeats)], where 1 indicates perfect heartbeat perception. To calculate interoceptive accuracy insight, the mean heartbeat perception score and confidence rating of each participant were linearly transformed into percent of maximum possible (POMP) scores^46^. This enables a direct comparison between the two variables by scaling both on a 0 to 100 scale. We then took the absolute difference between heartbeat perception scores and ratings and subtracted it from 100 so that higher scores indicate better interoceptive insight (i.e., a smaller discrepancy between performance and beliefs)^47^. After the HCT, participants were asked to rate their emotional experience during the task on the following dimensions^39^: valence (Self-Assessment Manikin; 1 *negative* to 9 *positive*)^48^, arousal (Self-Assessment Manikin; 1 *calm* to 9 *aroused*)^48^, anxiousness (1 *very anxious/worried* to 9 *not at all anxious/worried*); and to indicate their general confidence in their heartbeat perception (1 *very confident* to 9 *not at all confident*). We reverse-scored anxiousness and confidence so that in the results higher scores indicate higher anxiety and higher confidence.

### Physiological data

Data were recorded from actively shielded Ag/AgCl electrodes with a Refa amplifier and Polybench 1.3 software (TMSi, Twente Medical Systems International) at a sampling rate of 512 Hz. EEG was recorded from 63 equidistant electrodes with online average reference and a ground electrode placed on the left wrist. Impedances were kept below 10 kΩ. Horizontal EOG electrodes were attached to the outer canthi of the eyes. ECG electrodes were placed on the upper sternum and distal end of the left costal arch.

### Heart rate and heart rate variability

ECG data were analysed in ANSLAB 2.6^49^. Data were re-sampled at 400 Hz and bandpass filtered from 0.5 to 40 Hz with a 50 Hz notch filter. R peaks were detected automatically and manually edited, if necessary. Heartrate, HRV (fast-Fourier-transform-derived log HF power: 0.15–0.50 Hz), and exact numbers of R peaks per HCT counting interval were extracted.

### Heartbeat-Evoked potentials

EEG data were analysed in BrainVision Analyzer 2.2.1 (Brain Products). After down-sampling to 256 Hz, zero-phase-shift Butterworth filters were applied: a 0.1–35 Hz bandpass filter (half-power cutoff, 24 dB/octave) and a 50 Hz notch filter (± 2.5 Hz, 96 dB/octave). Major artefacts in the data were detected automatically, including muscle activity and non-physiological glitches, excepting eye movements and blinks. Ocular correction independent component analysis (ICA) was performed with Fp1 as VEOG channel, to remove eye blinks and eye movements. Contaminated or unavailable channels were excluded from ICA and interpolated afterwards (spherical splines). Data were re-referenced to a common average reference (included channels: 10L, 10R, 1LA, 1LB, 1LC, 1RA, 1RB, 1RC, 1Z, 2L, 2LA, 2LB, 2LC, 2R, 2RA, 2RB, 2RC, 2Z, 3L, 3LA, 3LB, 3R, 3RA, 3RB, 3Z, 4L, 4LB, 4LC, 4LD, 4R, 4RB, 4RC, 4RD, 5L, 5LB, 5LC, 5R, 5RB, 5RC, 5Z, 6L, 6R, 6Z, 7L, 7R, 7Z, 8L, 8R, 9L, 9R, Cz, Fp1, Fp2, Iz, Oz, T7, T8). R peaks were detected automatically in the ECG and manually edited, if necessary. Segments were created from −250 to 1000 ms around the R peaks. Segments containing artefacts, such as muscle activity, drifts, or non-physiological glitches, were detected and removed automatically. Averages were created for the resting period and the HCT, and baseline corrected from −250 to −50 ms, thus excluding the onset of the QRS complex^30^. We pooled across a large fronto-central cluster (1Z, 2Z, 3Z, 4Z(Cz), 1L(Fp1), 1R(Fp2), 1LB, 1RB, 2L, 2R, 3L, 3R, 4L, 4R, 2LB, 2RB, 1LA, 1RA, 3LB, 3RB, 5LA, 5RA, 3LA, 3RA, 2LA, 2RA, 4LB, 4RB, 1RC, 2RC, 3RC(T8), 3LC(T7), 1LC, 2LC, 5Z, 6 L, 6R, 6Z), as a recent meta-analysis located effects of attention, interoceptive performance, and clinical group in this scalp region^30^, including differences between patients with AN and HC^31^. Mean amplitudes were extracted for a time window of 455 to 595 ms after the R peak^31^, as the electric field artefact propagating from the heart to the scalp (cardiac field artefact) is minimal during this interval^50^. Nevertheless, a control analysis was performed on the ECG channel, to explore a potential contribution of differences in cardiac activity^26^. HEPs were not analysed for *n* = 7 participants (AN = 3, BN = 4), who met the a priori-defined threshold of 25% or more segments rejected due to artefacts^51^. The remaining participants had on average 332.67 (*SD* = 59.87) valid HEP segments for the resting condition and 351.37 (*SD* = 49.93) for the HCT.

### Statistical analysis

The diagnostic groups (HC vs. AN vs. BN vs. BED vs. OB) were compared with analyses of variance (ANOVAs) for interoceptive accuracy performance, beliefs, and insight. For HR, HF HRV, HEPs, and ECG the factor condition (rest vs. HCT) was added. Significant main effects or interactions were followed up with Bonferroni-corrected pairwise comparisons. ANOVA test statistics for all dependent variables are summarised in Table 4, while descriptive statistics are reported in Supplementary Table S1. Alpha was set to 0.05, effect sizes are reported as partial eta squared. Analyses were conducted with IBM SPSS Statistics v27.

## Results

### Sample characteristics

The groups did not differ significantly regarding years of education. The BED and OB groups were significantly older than the HC, AN, and BN groups. The AN group had the lowest BMI, followed by the HC and BN groups, then BED, and then OB with the highest BMI. Symptoms of anxiety were significantly elevated in the AN and BN groups and somewhat elevated in the BED group, who did not differ significantly from any other group. Regarding eating behaviour (DEBQ), the AN and BN groups showed significantly higher levels of restrained eating than the HC, BED, and OB groups. On the DEBQ emotional eating subscale, the HC and AN groups had the lowest scores, followed by OB, and then BN and BED with the highest scores. Regarding DEBQ external eating, the AN group had significantly lower scores than all other groups, who did not differ significantly from each other. Twenty-nine patients with AN were diagnosed with restrictive and nine with binge-purge subtype. Sociodemographic and clinical characteristics are summarized in Table 2 and comorbid disorders in Table 3.

**Table 2:** Sociodemographic and clinical characteristics.

Characteristic	HC *n* = 46	AN *n* = 38	BN *n* = 35	BED *n* = 22	OB *n* = 20	Test statistics
	*M* (*SD*)	*M* (*SD*)	*M* (*SD*)	*M* (*SD*)	*M* (*SD*)	*F* (*df*1, *df*2) *p*, η_p_^2^
Age	21.98 (3.45)^a^	23.03 (5.30)^a^	24.46 (7.72)^a^	34.23 (10.99)^b^	32.55 (7.33)^b^	19.06 (4, 156) < 0.001, 0.33
BMI	21.97 (1.68)^a^	15.43 (1.78)^b^	22.93 (3.84)^a^	31.11 (5.66)^c^	37.07 (6.08)^d^	138.64 (4, 156) < 0.001, 0.78
Years of education	14.69 (2.38)	14.96 (2.97)	14.07 (2.53)	15.32 (5.05)	14.68 (3.23)	0.63 (4, 154) 0.64, 0.016
STAI-T	40.83 (10.51)^a^	55.18 (9.32)^b^	56.31 (12.92)^b^	47.59 (12.42)^a, b^	41.55 (9.36)^a^	15.65 (4, 156) < 0.001, 0.29
DEBQ Restrained Eating	2.55 (0.80)^a^	3.72 (0.95)^b^	3.70 (0.86)^b^	3.06 (0.63)^a^	2.89 (0.62)^a^	15.51 (4, 156) < 0.001, 0.29
DEBQ Emotional Eating	2.32 (0.74)^a^	1.81 (1.14)^a^	3.95 (0.86)^b^	4.01 (0.56)^b^	3.22 (0.61)	45.22 (4, 156) < 0.001, 0.54
DEBQ External Eating	3.38 (0.54)^a^	2.56 (0.77)	3.65 (0.68)^a^	3.66 (0.62)^a^	3.56 (0.57)^a^	17.60 (4, 156) < 0.001, 0.31

Note. Groups with identical superscripts do not differ significantly (*p* <.05 in Bonferroni-corrected post-hoc test). HC = healthy control; AN = anorexia nervosa; BN = bulimia nervosa; BED = binge eating disorder; OB = obesity; BMI = body mass index; STAI-T = State-Trait Anxiety Inventory – Trait version; DEBQ = Dutch Eating Behaviour Questionnaire.

**Table 3:** Current comorbid mental disorders.

Disorder	HC *n* = 46	AN *n* = 38	BN *n* = 35	BED *n* = 22	OB *n* = 20
	*n* (%)	*n* (%)	*n* (%)	*n* (%)	*n* (%)
Anxiety disorder	0	11 (28.9)	16 (45.7)	13 (59.1)	10 (50.0)
Major depressive episode	0	20 (52.6)	12 (34.3)	4 (18.2)	1 (5.0)
PTSD	0	8 (21.1)	0	0	1 (5.0)
OCD	0	6 (15.8)	1 (2.9)	1 (4.5)	0
Bipolar disorder	0	1 (2.6)	1 (2.9)	1 (4.5)	0

Note. HC = healthy control; AN = anorexia nervosa; BN = bulimia nervosa; BED = binge eating disorder; OB = obesity; PTSD = post-traumatic stress disorder; OCD = obsessive-compulsive disorder.

### Heart rate and heart rate variability

**Fig. 1:**
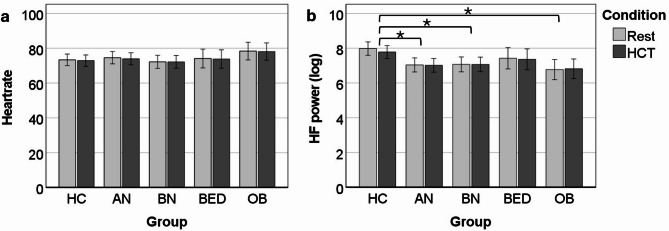
Group differences in heartrate (**a**) and heartrate variability (**b**; log HF power) by condition. There were no significant effects regarding heartrate. Heartrate variability was significantly lower in the AN, BN, and OB groups than in the HC groups. Error bars represent 95% CI. HF = high frequency heart rate variability; HCT = heartbeat counting task; HC = healthy control; AN = anorexia nervosa; BN = bulimia nervosa; BED = binge eating disorder; OB = obesity. * = post-hoc Bonferroni-corrected pairwise comparison significant at *p* <.05.

All ANOVA test statistics are reported in Table 4. For HR, there were no significant effects for group, condition, or their interaction. For HF power, there was a significant effect for group, but none for condition, or the interaction. The AN, BN, and OB groups had significantly lower HF power than the HC group (Fig. 1).

### Heartbeat-Evoked potentials

**Fig. 2:**
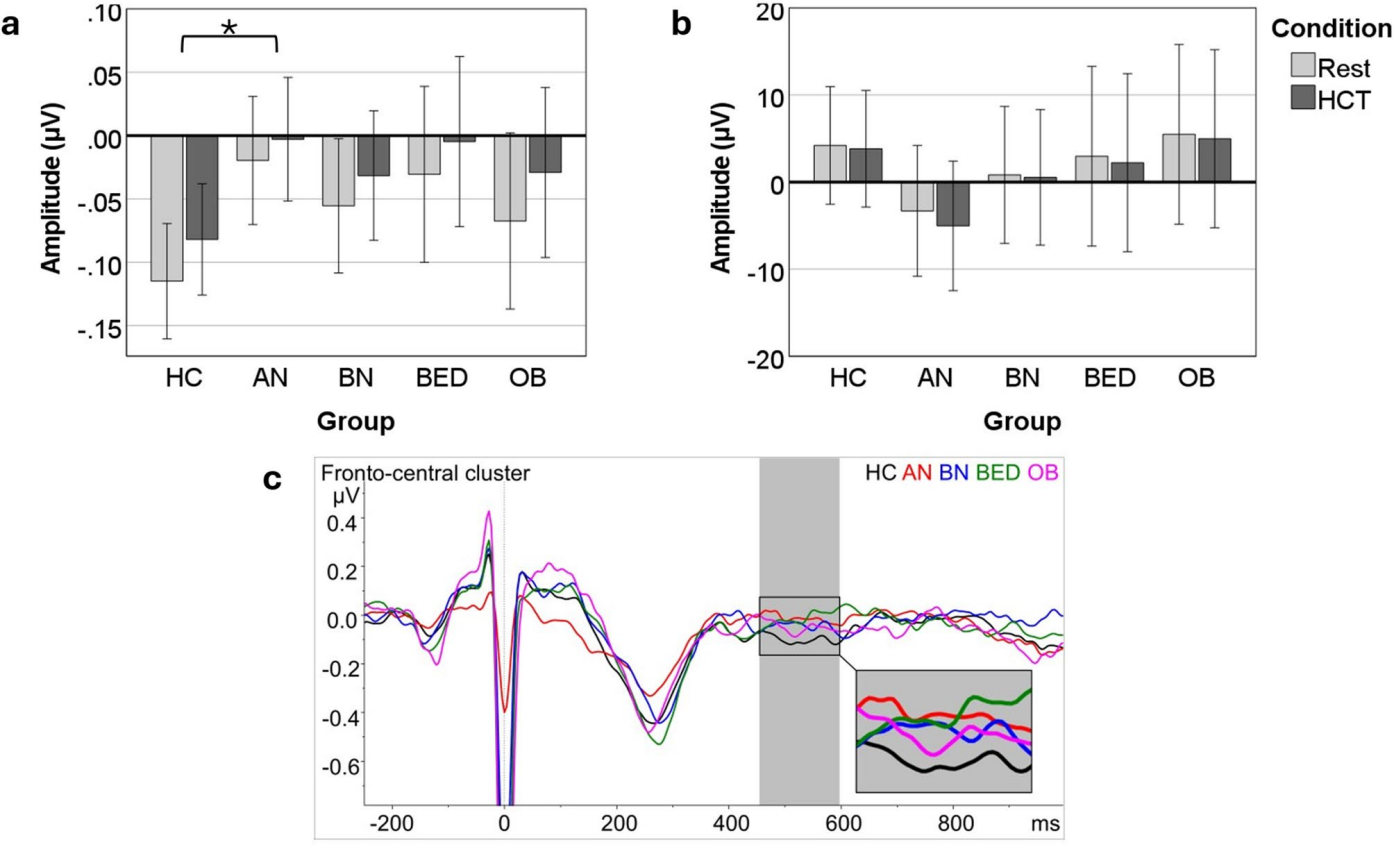
Group differences in heartbeat evoked potentials (HEPs) by condition. Panel a shows that the AN group had significantly higher HEPs than the control group and that HEPs were significantly more positive during the heartbeat counting task than during rest. Panel b shows non-significant effects on the ECG (control analysis). Panel c shows HEP waveforms, averaged across conditions and electrodes of the fronto-central cluster, using an average reference. The groups are displayed as separate lines. The time-window of interest (455–595 ms) is highlighted by a grey rectangle and magnified in the inlay. Error bars in Panels A and B represent 95% CI. HCT = heartbeat counting task; HC = healthy control; AN = anorexia nervosa; BN = bulimia nervosa; BED = binge eating disorder; OB = obesity. * = post-hoc Bonferroni-corrected pairwise comparison significant at *p* <.05.

There was a significant main effect for condition, with more positive HEPs during the HCT than during rest. The main effect for group was also significant. Post-hoc tests showed relatively more positive HEPs for the AN than for the HC group (*p* =.047; Fig. 2a). The interaction between condition and group was not significant. The control analysis on the ECG channel did not produce any significant effects for condition, group, or their interaction (Fig. 2b). Waveforms are shown in Fig. 2c.

### Heartbeat counting task

**Fig. 3:**
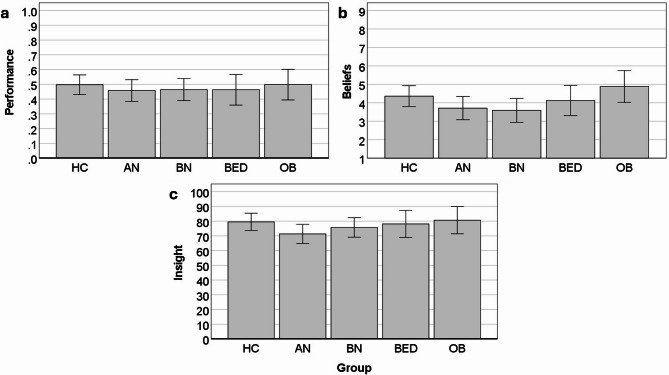
Group differences for interoceptive accuracy performance (**a**), beliefs (**b**), and insight (**C**), as assessed with the heartbeat counting task. There were no significant main effects. Error bars represent 95% CI. HC = healthy control; AN = anorexia nervosa; BN = bulimia nervosa; BED = binge eating disorder; OB = obesity.

**Fig. 4:**
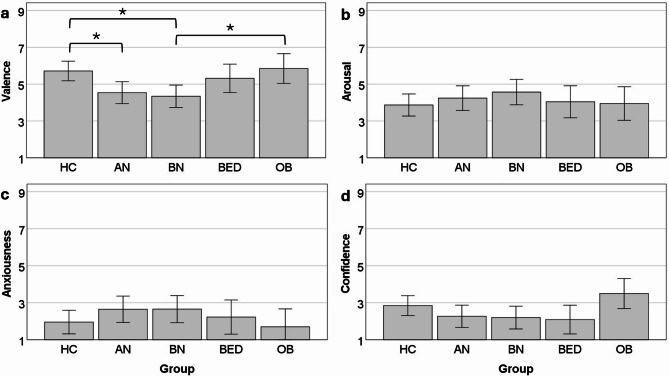
Group differences for emotional evaluation of the heartbeat counting task. Panel a shows valence ratings (1 = negative, 9 = positive), where the AN and BN groups had significantly more negative ratings than the HC group, and the BN group had significantly more negative ratings than the OB group. Panel b shows arousal ratings (1 = low, 9 = high), where no significant group effect was found. Panel c shows anxiousness ratings (1 = not at all anxious, 9 = very anxious), also with a non-significant group effect. Panel d shows ratings for overall confidence in one’s performance during the heartbeat counting task (1 = not at all confident, 9 = very confident), where a significant main effect for group was found, but none of the post-hoc tests reached statistical significance. Error bars represent 95% CI. HC = healthy control; AN = anorexia nervosa; BN = bulimia nervosa; BED = binge eating disorder; OB = obesity. * = post-hoc Bonferroni-corrected pairwise comparison significant at *p* <.05.

There were no significant group differences for interoceptive accuracy performance, beliefs, or insight (Fig. 3). Concerning the emotional evaluation of the HCT, the groups differed significantly regarding the experience of negative/positive affect during the HCT. The AN (*p* =.041) and BN (*p* =.010) groups experienced the task more negatively than the HC group, and the BN group experienced the task more negatively than the OB group (*p* =.038; Fig. 4). The groups also differed significantly regarding their general confidence in their performance, though none of the post-hoc tests reached statistical significance. Descriptively, the OB group had the highest confidence in their performance, followed by the HC group and then the three ED groups with the lowest confidence. There were no significant differences for arousal or anxiousness during the HCT.

**Table 4:** ANOVA test statistics for all dependent variables.

Dependent variable	Effect	df1, df2	F	*p*	η_*p*_^2^
HR	Group	4, 141	1.02	0.40	0.028
	Condition	1, 141	1.83	0.18	0.013
	Group × Condition	4, 141	0.93	0.93	0.006
HRV	Group	4, 140	4.02	0.004	0.10
	Condition	1, 140	0.93	0.34	0.007
	Group × Condition	4, 140	0.87	0.48	0.024
HEP	Group	4, 138	2.45	0.049	0.066
	Condition	1, 138	5.82	0.017	0.040
	Group × Condition	4, 138	0.12	0.98	0.003
ECG	Group	4, 138	0.89	0.47	0.025
	Condition	1, 138	0.69	0.41	0.005
	Group × Condition	4, 138	0.12	0.98	0.003
Accuracy performance	Group	4, 146	0.24	0.91	0.007
Accuracy beliefs	Group	4, 155	1.99	0.099	0.049
Accuracy insight	Group	4, 146	1.10	0.36	0.029
Valence	Group	4, 155	4.60	0.002	0.11
Arousal	Group	4, 155	0.66	0.62	0.017
Anxiety	Group	4, 155	1.12	0.35	0.028
Confidence	Group	4, 155	2.49	0.045	0.060

Note. HR = heartrate; HRV = heartrate variability (log high frequency power); HEP = heartbeat evoked potential; ECG = electrocardiogram.

### Exploratory correlational analyses

On an exploratory level, we performed Pearson correlations between dependent and clinical variables (BMI, DEBQ subscales, STAI-T), see Supplementary Table S3. There was a significant positive correlation between HEPs and STAI-T. HEPs during rest, but not during the HCT were related to HR (positively) and HRV (negatively). HEPs did not correlate significantly with any other dependent or clinical variable. More negative affect and higher anxiety during the HCT were associated with poorer performance and more pessimistic beliefs in the HCT, higher DEBQ restrained eating scores, and higher trait anxiety (STAI-T). Trait anxiety correlated negatively with BMI and positively with emotional and restrained eating (DEBQ).

## Discussion

The aim of the present study was to establish interoceptive profiles for eating- and weight disorders, using a range of interoceptive facets regarding the perception of cardiovascular signals. Participants with obesity showed lower HRV (peripheral signal) than the HC group, but no alterations in any other interoceptive facet. Participants with AN and BN showed reduced HRV as well, but, in addition, reported prominent negative emotions during interoceptive focus (HCT). Only the AN group showed increased CNS processing of cardio-afferent signals. The BED group did not show any marked alterations.

### Anorexia and bulimia nervosa

#### Central-Nervous system processing

Our finding of increased CNS processing of cardioafferent signals in AN, but not BN, is in line with our previous results^31, 32^. A recent report found several temporo-topographical clusters of increased and decreased HEP activity in adolescent patients with AN, indicating that the picture might be more complex, although methodological differences limit the comparability between studies^33^. Supporting increased CNS processing, neuroimaging studies showed increased activation in the right anterior insula in AN during heartbeat perception^52^, a brain area which has been proposed as one of the potential sources of HEPs^53^. Other studies showed intensified interoception in AN especially for the anticipation of aversive stimuli, such as increased insula response during food image^54^ and pain anticipation^55^, and increased cardiac sensations during meal anticipation^56^.

It has been proposed in the literature that fasting might serve to suppress negatively evaluated body sensations^11^. Both individuals with AN and BN report being highly sensitive to sensory input and avoiding it more than healthy individuals do^57, 58^. This has been linked to body dissatisfaction^58^ and self-disgust^57^. Similar findings have been reported for the gastro-intestinal system, with self-reported sensitivity to gastro-intestinal sensations being related to body dissatisfaction and weight-loss behaviours across EDs^59^. The global picture is thus one of a general rejection of the body and all sensations associated with it, which potentially leads to body dissatisfaction and dietary restraint. The causality of the effect, however, remains to be determined.

#### Emotional evaluation

Both, individuals with AN and BN, engage in dietary restriction and other weight-loss behaviours, and both groups evaluated heartbeat perception negatively in our study, but only those with AN showed increased CNS processing. Negative evaluations, thus, do not appear to be driven by increased CNS processing, but to represent a distinct facet of interoception, further highlighting the need to distinguish between facets. We would argue that the combination of increased CNS processing plus negative evaluation supports more extreme dieting behaviours in AN than BN. Of note, the majority of our sample with AN was diagnosed with the restrictive subtype. Exploratory correlations confirmed that negative evaluation was associated with restrained eating, but not emotional or external eating. We need to stress, however, that the current data are cross-sectional in nature and that negative evaluation of cardioceptive focus may be an expression of general negative affect and anxiety associated with an acute mental disorder, as supported by the correlation between valence ratings and trait anxiety. Future research should elucidate the mechanisms linking the neural processing of interoceptive information with the affective evaluation thereof and ED symptoms, clarify the role of trait anxiety, and include longitudinal investigations to allow for causal inferences. Comparing patients with restrictive and binge-purge subtypes of AN with those with BN may help determine the role of restrictive psychopathology.

#### Peripheral organ activity

Our finding of reduced HF HRV in individuals with AN and BN contrasts with recent systematic reviews documenting increased HF in these groups^21, 22^. Patients in our study were undergoing intensive psychosomatic inpatient treatment, which included eating three meals per day (~ 2100 kcal) during refeeding. Vagal dominance appears to be linked to an acute fasting state in individuals with BN^23^. It decreases with treatment/refeeding in AN^22^ and in BN, though results are mixed^21^. Thus, reduced HRV in AN and BN in our sample may be linked to the specific treatment context. In addition, many patients in our sample reported comorbid anxiety or major depressive disorders, both associated with reduced HRV^60, 61^.

#### Interoceptive accuracy performance, beliefs, and insight

Despite these alterations in peripheral, neural, and affective processing, neither patients with AN nor BN showed significant alterations in interoceptive accuracy performance, beliefs, or insight. As findings of previous studies are mixed, it is possible that effects depend on factors such as sample characteristics or state variables. Cardioceptive alterations in AN, for example, have been linked to anxiety and the meal context^62^. Our results suggest that notwithstanding the proposed rejection of bodily sensations, patients with AN and BN are able to count their own heartbeats just as well as healthy women, when explicitly prompted to do so. This may be different from a repression of bodily sensations in everyday life, where eating pathology is associated with distraction from and a lack of trust in interoceptive signals^63^.

#### Interoceptive profiles and clinical implications

In summary, rather than showing a global interoceptive deficit, patients with AN presented with reduced parasympathetic cardiac modulation, increased CNS processing, unaltered interoceptive accuracy performance, beliefs, and insight, and increased negative evaluation of heartbeat perception. Patients with BN showed no significant alterations in CNS processing but an otherwise similar profile. Amplified CNS processing supports the use of interoceptive exposure in the treatment of AN^13^. Additionally, HEP-based neurofeedback could be explored in this context, although it remains challenging due to the HEP’s poor signal-to-noise ratio, underscoring the importance of finding alternative markers of heart–brain coupling suitable for neurofeedback^64^. Both patients with AN and BN might benefit from cognitive interventions targeting the negative evaluation of interoceptive stimuli.

### Binge eating disorder and obesity

Our finding of reduced HF HRV in obesity is consistent with previous results^20^. We could not replicate meta-analytic findings of reduced interoceptive accuracy performance, however^35^. Note that the overall effect size of the five studies reporting OB-HC comparisons included in this meta-analysis was small (SMD = − 0.389), and two of the studies included only children. Thus, our OB sample may have been too small to detect the effect. At a descriptive level, however, the HC and OB samples in our study had virtually identical heartbeat perception accuracy performance scores. It is possible, therefore, that differences in sample characteristics (e.g., children vs. adults, overweight vs. OB) and task instructions (i.e., instructing participants not to guess the number of heartbeats) to other studies can account for the diverging results. Patients with BED did not differ significantly from the other groups on any of the variables related to cardiac interoception.

Overall, neither the group with OB nor the group with BED showed marked interoceptive alterations. It appears, therefore, that interoceptive alterations regarding the *cardio-vascular system* might be more related to fasting and compensatory behaviour than to overeating. This is confirmed by our exploratory correlational analyses, where external and emotional eating were *not* significantly related to HEPs, interoceptive accuracy, or emotional evaluation, whereas restrained eating *was* related to emotional evaluation. Similarly, non-acceptance of emotions has been linked to dietary restraint rather than binge eating^65^. It is possible that overeating, in contrast, is primarily related to interoceptive alterations regarding the *gastro-intestinal system*, especially difficulties in perceiving stomach fullness and alterations in gastric myoelectrical activity^6^. Difficulties distinguishing between hunger and emotions also appear to be linked particularly to conditions involving binge eating (i.e., BN and BED)^7^. Based on our results, it seems unlikely that these difficulties are caused by a deficit in cardiac interoception.

## Limitations

Less than optimal sample sizes for the BED and OB groups limit the interpretability of the data, especially considering the small effect size reported for interoceptive accuracy in OB in a recent meta-analysis^35^. Yet, in the present study AN, BN and HC samples were of decent size and larger than in many similar experimental studies included in recent systematic reviews^4^. Effects of psychotropic medication on interoception remain underexplored and unclear, yet preliminary evidence suggests selective serotonin reuptake inhibitors might improve cardiac interoceptive accuracy^66^. Since information on medication status was unavailable for the current sample, we cannot exclude that this may have affected our results. There is an ongoing debate in the literature regarding the validity of the HCT for the assessment of interoceptive accuracy performance, with some researchers and studies opposing and others supporting its validity; see^67^ for a detailed discussion. Other tasks have been proposed, which overcome some of the potential shortcomings of the HCT, e.g., the heartbeat discrimination task^68^. Future research on cardioception in EWDs should include alternative tasks in a ‘multi-measure’ approach. One advantage of the HCT, however, is that it induces an interoceptive focus, which allowed us to uncover negative emotional evaluations of heartbeat sensations in AN and BN.

## Conclusion

In contrast to the general interoceptive deficit often proposed in the literature, we found only few alterations in cardiac interoception in EWDs, despite reduced vagal tone in AN, BN, and OB. Noteworthy findings concern the negative evaluation of cardioceptive sensations in AN and BN, as well as increased HEPs in AN. Future research should investigate how fasting and other compensatory behaviours might serve to alleviate negatively evaluated and potentially enhanced interoceptive signals. In addition, intervention techniques targeting these specific alterations, e.g., interoceptive exposure^13^, should be further explored. The results of the current study underline the need to assess multiple facets of interoception in concurrence. Additionally, future studies should examine different bodily systems in EWDs, especially the gastrointestinal system.

## Data Availability

The datasets generated and analysed during the current study are available on the OSF repository, 10.17605/OSF.IO/QZ4YB.
